# Morselized Bone Graft: A Tool for Nasal Dorsum Refinement and Camouflaging

**DOI:** 10.1093/asjof/ojaf147

**Published:** 2025-11-11

**Authors:** Shaishav Datta, Buğra Tugertimur, Alexia Lucas, Alannah Phelan, Matthew Morris, Paige Goote, Richard Westreich, Steven A Hanna, David Mattos, Richard G Reish

## Abstract

**Background:**

Refining the nasal dorsum to achieve a smooth and natural contour remains challenging, particularly in patients with thin skin who are prone to visible surface irregularities. Numerous techniques have been described to address these issues, including diced cartilage, fascial or dermal grafts, and synthetic implants.

**Objectives:**

This study evaluates the outcomes of using morselized bone grafts (MBG), specifically, autologous bone rasp material that is typically discarded, as a method for nasal dorsum contour refinement.

**Methods:**

A retrospective review was conducted of consecutive rhinoplasty procedures performed by the senior author between January 2021 and June 2022. Patients who underwent dorsal contouring with MBG and had at least 12 months of follow-up were included. The primary outcomes were postoperative infection and the need for revision surgery.

**Results:**

A total of 953 patients met inclusion criteria. The mean patient age was 31.6 ± 11.3 years, and the mean follow-up duration was 23.5 ± 8.7 months. Postoperative infections occurred in 26 patients (2.7%), all of which resolved with antibiotic therapy. Sixteen patients (1.7%) required operative revision.

**Conclusions:**

The use of MBG harvested from bone rasp material provides a safe and efficient option for achieving dorsal nasal smoothness and camouflaging minor contour irregularities in both primary and revision rhinoplasty. Additionally, MBG use is an efficient alternative to other techniques for addressing dorsal esthetics, specifically camouflaging minor irregularities, with no additional donor-site morbidity when paired with boney dorsal reduction.

**Level of Evidence: 4 (Therapeutic):**

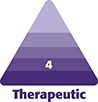

The nasal dorsum is a cornerstone of nasal esthetics, playing a vital role in achieving facial harmony and balance. Achieving a smooth, refined nasal profile remains a significant challenge, particularly in thin-skinned patients who are more susceptible to contour irregularities. Many techniques are used to address this problem, including cartilage, fascia, acellular dermal matrices (ADM), silicone implants, and lipofilling.^1-5^

When considering autologous options, there is often a paucity of septal cartilage in revision cases. Further, when cartilage is available, it is often diced and used in conjunction with fascia or ADM, adding both donor site morbidity and increased operative time to each case.^4,5^ Fascial grafts alone from various sources, for example, deep mastoid fascia, rectus abdominis fascia, and temporal fascia, have all been used; however, these add another donor site and increase length of surgery. Allograft options such as ADM can be expensive and are associated with high rates of resorption.^4,6^ Silicone implants are associated with devastating esthetic complications such as nasal capsular contracture, implant dislocation, implant extrusion, permanent skin thickening, and color changes.^4,7^ Finally, micro-fat injections offer advantages over other options as they can be performed in an ambulatory setting.^5,8^ However, lipofilling is unpredictable due to high rates of fat resorption. Some of the abovementioned techniques also lead to dorsal augmentation, whether intended or inadvertently. Further, the micro-irregularities of the dorsum may persist if there is any roughness to the grafts. Thus, the ideal nasal dorsal contour graft helps camouflage contour minor irregularities while providing esthetically pleasing and robust outcomes with minimal complications or need for revision. Further, any autologous options should have minimal donor site morbidity.

In this study, the effectiveness of morselized bone grafts (MBG)—specifically, unused bone rasp material that is typically discarded—as a technique for contouring and refining the nasal dorsum after dorsal reduction is evaluated. This technique is versatile in that it can be used as an adjunct with any of the already employed techniques to address irregularities in nasal dorsum contour.

## METHODS

Retrospective chart review of the senior author's practice for all cases of consecutive rhinoplasty conducted between January 2021 and June 2022 was performed. The study was approved by the Research Ethics Board at the Biomedical Research Alliance of New York. All patients whose photographs were used in the study provided consent.

The inclusion criteria for the study included any patients who underwent open rhinoplasty where MBG was utilized for nasal dorsum refinement. All patients who underwent rhinoplasty, including primary and revision cases, with a minimum of 12 months of follow-up were included in the study.

Full chart review of all patients who met inclusion criteria was conducted to collect demographic data and surgical outcomes including review of all follow-up encounters. Clinical examination and palpation at time of follow-up were used to assess for persistent nasal dorsum irregularities. All patients received a course of prophylactic antibiotics in the immediate postoperative period, either cefadroxil or clindamycin for 7 days, based on patient allergies. Postoperative infections included any cases in which patients demonstrated clinical signs of infection requiring further antibiotics or any form of surgical intervention. Any patient who required subsequent open rhinoplasty was included in our measure of rate of revision. The primary outcomes in our study were rate of infection and rate of revision.

### Statistical Analysis

Descriptive statistical analyses were performed to summarize patient characteristics and surgical outcomes. Continuous variables, including age and follow-up duration, are reported as mean ± standard deviation and range. Categorical variables, such as sex, type of rhinoplasty (primary vs revision), incidence of postoperative infection, and rate of operative revision, are reported as frequencies and percentages. No inferential or comparative statistical tests were performed, as the study was retrospective and primarily descriptive in nature. All data were analyzed using Microsoft Excel (Microsoft Corp., Redmond, WA), and percentages were calculated based on the total number of patients within each relevant subgroup.

### Surgical Technique

All cases were performed under a general anesthetic with an open rhinoplasty approach through a transcolumellar incision. After performing a dorsal hump reduction with a bone rasp, the MBG is collected from the rasping instrument and stored on the back table in saline for later use in the case. The raspings are then dehydrated on a sheet of Telfa to create the final MBG paste. After addressing other components of the rhinoplasty procedure including any osteotomies, cartilage grafts for the middle third/nasal dorsum, and nasal tip positioning and refinements, attention is turned back to the nasal dorsum, wherein any contour irregularities at the keystone region or middle third of the nasal dorsum are filled with the MBG paste. On average, approximately 0.25 cc of MBG is applied to the nasal dorsum. The MBG placed using bayonet forceps and is gently packed and molded with external pressure until the desired contour is achieved; any excess material that protrudes beyond the intended surface is discarded before closure. Positioning is confirmed with the skin re-draped, ensuring that any dorsal irregularities have been addressed. The operative technique is demonstrated in Video 1.

## RESULTS

**Figure 1.**
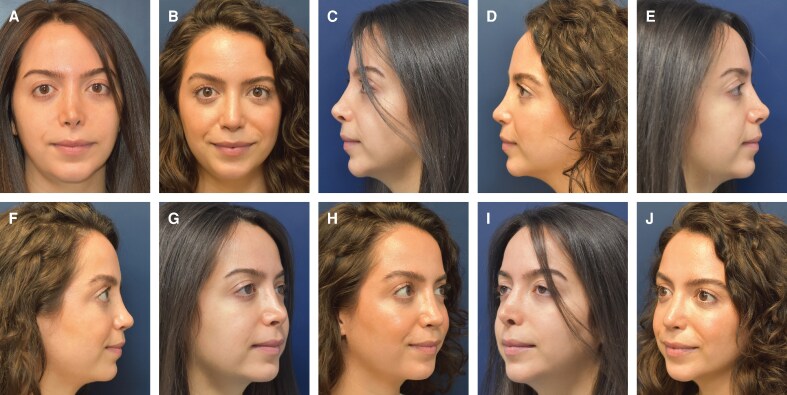
A 34-year-old woman is shown preoperatively (A, C, E, G, I) and 1-year postoperatively (B, D, F, H, J). This patient had a previous rhinoplasty which left her with a pollybeak deformity, thin dorsal skin, deviated mid-vault with collapse, tip asymmetry, nostril asymmetry, over-projection of the tip, and a foreshortened nasal tip with over-exposure of her nostrils. The senior author performed revision rhinoplasty with dorsal hump reduction, placement of columellar strut and extended spreader grafts with cadaveric fresh frozen costal cartilage to add tip support, correction of nasal tip asymmetry, correction of mid vault collapse, decrease in nostril exposure, tip derotation, tip deprojection, hybrid cartilage, and fascia tip graft to unify and refine her tip, and placement of morselized bone dorsal onlay graft to help smooth the dorsal contour over her thin skin.

**Figure 2.**
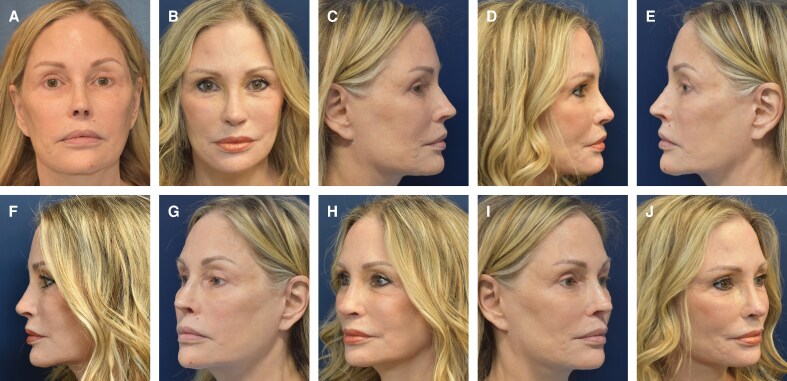
A 65-year-old woman is shown preoperatively (A, C, E, G, I) and 1-year postoperatively (B, D, F, H, J). This patient had 3 previous rhinoplasties, which left her with thin dorsal skin, a dorsal hump, tip asymmetry, nostril asymmetry, over-projection of the tip, and an extremely foreshortened nasal tip with over-exposure of her nostrils. The senior author performed revision rhinoplasty with dorsal hump reduction, placement of columellar strut and extended spreader grafts with cadaveric fresh frozen costal cartilage to add tip support, correction of nasal tip asymmetry, decrease in nostril exposure, tip derotation, tip deprojection, hybrid cartilage and fascia tip graft to unify her tip, and finally placement of morselized bone dorsal onlay graft to help smooth the dorsum.

There were 953 patients who met criteria and were included in the study (869 female patients and 84 male patients) with a mean age of 31.6 ± 11.3 years old (range, 14-89 years old). Six hundred and seventy-two patients underwent primary rhinoplasty. There was a minimum of 1 year of follow-up with a mean of 23.5 ± 8.7 months (range, 12-40 months). The postoperative infection rate was 2.7%, with 26 patients requiring postoperative antibiotics. 16 (1.7%) patients required operative revision, among which 11 (68.8%) were revision rhinoplasty cases. There were no cases of postoperative infection that required acute surgical management. There were no patients who sought revision surgery for concerns related to dorsal irregularities or contour defects. Table 1 provides a summary of demographic data for the patients in the study. Figure 1 and Video 2 and Figure 2 and Video 3 show examples of 2 patients who underwent rhinoplasty with application of MBG after dorsal hump reduction for nasal dorsum refinement.

**Table 1.** Patient Demographics

Patient demographics	
Sex	
Female	869 (91.2%)
Male	84 (8.8%)
Age	
Mean ± SD	31.6 ± 11.3 yr
Range	14-89 yr
Follow-up	
Mean ± SD	23.5 ± 8.7 mo
Range	12-40 mo

Patient demographics	
Primary	672 (70.5%)
Revision	281 (29.5%)
Complication rates	
Infection	26 (2.7%)
Primary	16 (2.4%)
Revision	10 (3.6%)
Any revision surgery	16 (1.7%)
Primary	5 (0.7%)
Revision	11 (3.9%)

## DISCUSSION

This study assesses the complications of our technique using MBG for nasal dorsum contouring of minor irregularities, with a minimum of 1 year of follow-up. We provide an easy and reliable technique for addressing minor nasal dorsal contour irregularities to create a smooth and esthetic nasal dorsum with no additional donor site.

The senior author's practice is primarily focused on rhinoplasty, with a large proportion of these patients seeking revision rhinoplasty.^9,10^ Often, these patients have thin skin from repeated elevation of dorsal skin for open rhinoplasty approaches.^11-15^ The resultant thin skin leads to several potential complications, chiefly, visibility of the underlying structures and associated irregularities, which can detract from the overall esthetic outcome.^12^ Depending on the type of underlying graft, there is possibility for tombstone deformity if solid graft is used, or extrusion if silicone implants are used. Even if a softer graft is used, such as diced cartilage wrapped in fascia or fascia alone, minor irregularities in graft shape can show through over time once swelling settles. Lipofilling can be useful in some instances; however, it is fraught with high rates of resorption, and if utilized on a patient with many revisions, often does not take well given the abundant scar tissue and abnormal tissue planes. Use of injectable fillers has gained popularity given its low cost and nonsurgical nature; however, for the same reasons, lipofilling does not work, filler to the nasal dorsum in a revision rhinoplasty patient can be challenging and unreliable. Further, the results are not permanent and require repeat injections. MBG is a cost-effective technique for addressing minor contour irregularities and camouflaging the nasal dorsum through application of a paste, similar to applying a putty to fill in small holes and cracks and smoothen drywall. Given it is a paste, structural support is needed to address any major nasal dorsum irregularities after which MBG can be utilized as an on-lay graft for further refinement. MBG volume was not standardized but rather dictated by the extent of rasping and contour irregularity present. The mechanism by which MBG maintains long-term nasal contour was not studied; however, we expect that the MBG is resorbed and replaced with a layer of scar, adding additional thickness to a thin-skinned nasal dorsum.

The rate of postoperative infection in this study was 2.7%, with all cases resolving with antibiotic treatment, not requiring further operative intervention.^16^ Of note, all infections occurred at either the columella or the nasal tip, there were no patients who endorsed signs and symptoms of infection over the nasal dorsum. The infection rate was higher in the revision rhinoplasty group when comparing infection rate within subgroups of primary (2.4%) and revision (3.6%) rhinoplasty. The same was found for rate of revision, wherein overall rate of revision was 1.7%, and the rate of operative revision was higher in the revision (3.9%) rhinoplasty group vs primary (0.7%) rhinoplasty. Importantly, no formal statistical comparison was performed between primary and revision rhinoplasty subgroups; therefore, observed differences in infection and revision rates are descriptive and should be interpreted cautiously. However, it is well understood that revision surgery results in the formation of scar tissue and fibrosis, poorer blood supply, and overall compromised quality of the soft tissue envelope, all of which leads to increased risk for infection and further operative revision.^16,17^

The nasal dorsum contour was assessed clinically by the senior author at patient follow-up appointments as well as asking patients to describe their cosmetic outcomes through feedback. Postoperative imaging was not routinely performed in this series. Further, intraoperative swelling may obscure subtle irregularities and that MBG resorption likely varies between patients. While clinical outcomes were favorable, future work should include validated esthetic outcome scales, standardized imaging assessments, and patient-reported outcome measures. Another limitation of the study is the inherent bias of retrospective studies, in which rates of revision are underreported, as it is possible for some patients to seek revision surgeries at other practices without our knowledge. Although the presence of minor palpable nasal dorsal irregularities was anecdotally higher prior to implementation of the MBG technique, this cannot be assessed retroactively, representing another limitation of the retrospective nature of this study. Finally, although a minimum of 1-year follow-up was used as an inclusion criterion, the nasal dorsum continues to heal at this time and assessment at the 1-year mark may not reflect the long-term revision rate. Nevertheless, we believe that the technique presented is novel and offers valuable insights, while recognizing that longer-term studies are necessary to fully assess durability and refine outcome expectations.

## CONCLUSIONS

This retrospective case series demonstrates the use of MBG for nasal dorsum refinement in both primary and revision rhinoplasty. In our study's group of 953 patients with at least 1 year of follow-up, the utilization of MBG for nasal dorsum esthetics was associated with revision rates comparable to the literature for both primary and revision rhinoplasty patients. MBG use is an efficient alternative to other techniques to camouflage minor dorsal irregularities with no additional donor-site morbidity when paired with boney dorsal reduction.

## References

[1] Zholtikov V, Golovatinskii V, Ouerghi R, Daniel RK. Rhinoplasty: aesthetic augmentation with improvement of dorsal aesthetic lines. Aesthet Surg J. 2021;41:759–769. doi: 10.1093/asj/sjaa345 33674857

[2] Wright JM, Halsey JN, Rottgers SA. Dorsal augmentation: a review of current graft options. Eplasty. 2023;23:e4. PMID: 36817363; PMCID: PMC9912050. 36817363 PMC9912050

[3] Jang YJ, Yoo SH. Dorsal augmentation in facial profiloplasty. Facial Plast Surg. 2019;35:492–498. doi: 10.1055/s-0039-1695726 31639874

[4] Lee MR, Unger JG, Rohrich RJ. Management of the nasal dorsum in rhinoplasty: a systematic review of the literature regarding technique, outcomes, and complications. Plast Reconstr Surg. 2011;128:538e–550e. doi: 10.1097/PRS.0b013e31822b6a82 22030516

[5] Daoud RM, Alelyani AA, Bakhamees BH, et al Thin skin in rhinoplasty: considerations for camouflaging dorsal nasal irregularities. Cureus. 2024;16:e66595. doi: 10.7759/cureus.66595 39258084 PMC11383643

[6] Tang Y, Han X, Li J, Cao Y, Gong Y, Xu C. A systematic review of technique, satisfaction, and complications of acellular dermal matrix in rhinoplasty. J Craniofac Surg. 2024;35:e250–e254. doi: 10.1097/SCS.0000000000010000 38345943

[7] Kim YK, Shin S, Kang NH, Kim JH. Contracted nose after silicone implantation: a new classification system and treatment algorithm. Arch Plast Surg. 2017;44:59–64. doi: 10.5999/aps.2017.44.1.59. Erratum in: Arch Plast Surg. 2017 Nov;44(6):575-576. doi: 10.5999/aps.2017.44.1.59. 28194349 PMC5300926

[8] Cárdenas JC, Carvajal J. Refinement of rhinoplasty with lipoinjection. Aesthetic Plast Surg. 2007;31:501–505. doi: 10.1007/s00266-006-0136-2 17653684

[9] Bagal AA, Adamson PA. Revision rhinoplasty. Facial Plast Surg. 2002;18:233–244. doi: 10.1055/s-2002-36491 12524595

[10] Spataro E, Piccirillo JF, Kallogjeri D, Branham GH, Desai SC. Revision rates and risk factors of 175 842 patients undergoing septorhinoplasty. JAMA Facial Plast Surg. 2016;18:212–219. doi: 10.1001/jamafacial.2015.2194 26967651 PMC5600890

[11] Rettinger G. Risks and complications in rhinoplasty. GMS Curr Top Otorhinolaryngol Head Neck Surg. 2007;6:Doc08. PMID: 22073084; PMCID: PMC3199839. 22073084 PMC3199839

[12] Tran KN, Jang YJ. Incidence and predisposing factors of postoperative infection after rhinoplasty: a single surgeon's 16-year experience with 2630 cases in an east Asian population. Plast Reconstr Surg. 2022;150:51e–59e. doi: 10.1097/PRS.0000000000009202 35511054

[13] Datta S, Tugertimur B, Hanna SA, et al Nasal tip deprojection in rhinoplasty. Plast Reconstr Surg. 2025; 155:439–444. doi: 10.1097/PRS.0000000000011697 39212968

[14] Tugertimur B, Datta S, Goote P, et al Mastoid fascia tissue graft as a tip camouflage technique in rhinoplasty: a reliable alternative to soft cartilage grafts. Plastic & Reconstructive Surgery. 2025;155:255–262. doi: 10.1097/PRS.0000000000011648 39026388

[15] Datta S, Mattos D, Hanna SA, Reish RG. Does soaking fresh frozen costal cartilage in an antibiotic solution reduce postoperative infection in rhinoplasty? Plast Reconstr Surg Glob Open. 2024;12:e5997. doi: 10.1097/GOX.0000000000005997 39036598 PMC11259397

[16] Neaman KC, Boettcher AK, Do VH, et al Cosmetic rhinoplasty: revision rates revisited. Aesthet Surg J. 2013;33:31–37. doi: 10.1177/1090820X12469221 23277618

[17] Hanna SA, Mattos D, Datta S, Reish RG. Outcomes of the use of fresh-frozen costal cartilage in rhinoplasty. Plast Reconstr Surg. 2024;154:324–328. doi: 10.1097/PRS.0000000000011125 37815290

